# Working memory training impacts the mean diffusivity in the dopaminergic system

**DOI:** 10.1007/s00429-014-0845-2

**Published:** 2014-07-15

**Authors:** Hikaru Takeuchi, Yasuyuki Taki, Rui Nouchi, Hiroshi Hashizume, Atsushi Sekiguchi, Yuka Kotozaki, Seishu Nakagawa, Carlos Makoto Miyauchi, Yuko Sassa, Ryuta Kawashima

**Affiliations:** 1 Division of Developmental Cognitive Neuroscience, Institute of Development, Aging and Cancer, Tohoku University, 4-1 Seiryo-cho, Aoba-ku, Sendai, 980-8575 Japan; 2 Division of Medical Neuroimaging Analysis, Department of Community Medical Supports, Tohoku Medical Megabank Organization, Tohoku University, Sendai, Japan; 3 Department of Radiology and Nuclear Medicine, Institute of Development, Aging and Cancer, Tohoku University, Sendai, Japan; 4 Human and Social Response Research Division, International Research Institute of Disaster Science, Tohoku University, Sendai, Japan; 5 Department of Functional Brain Imaging, Institute of Development, Aging and Cancer, Tohoku University, Sendai, Japan; 6 Smart Ageing International Research Center, Institute of Development, Aging and Cancer, Tohoku University, Sendai, Japan; 7 Graduate School of Arts and Sciences, The University of Tokyo, Tokyo, Japan

**Keywords:** Working memory, Training, Plasticity, Mean diffusivity, Dopamine, MRI, Diffusion-weighted imaging

## Abstract

Dopaminergic transmission plays a critical role in working memory (WM). Mean diffusivity (MD) is a sensitive and unique neuroimaging tool for detecting microstructural differences particularly in the areas of the dopaminergic system. Despite previous investigation of the effects of WM training (WMT) on dopamine receptor binding potentials, the effects of WMT on MD remain unknown. In this study, we investigated these effects in young adult subjects who either underwent WMT or received no intervention for 4 weeks. Before and after the intervention or no-intervention periods, subjects underwent scanning sessions in diffusion-weighted imaging to measure MD. Compared with no intervention, WMT resulted in an increase in MD in the bilateral caudate, right putamen, left dorsolateral prefrontal cortex (DLPFC), right anterior cingulate cortex (ACC), right substantia nigra, and ventral tegmental area. Furthermore, the increase in performance on WMT tasks was significantly positively correlated with the mean increase in MD in the clusters of the left DLPFC and of the right ACC. These results suggest that WMT caused microstructural changes in the regions of the dopaminergic system in a way that is usually interpreted as a reduction in neural components.

## Introduction

Working memory (WM) is the limited capacity storage system involved in the maintenance and manipulation of information over a short time period (Baddeley 2003). It underlies a wide range of higher-order cognitive activities such as reasoning and processing speed (Baddeley 2003; Osaka and Nishizaki 2000; Wechsler 1997).

Dopaminergic transmission plays a critical role in WM. Cortical dopamine release has been observed during WM (Aalto et al. 2005), and dopaminergic neurotransmission as well as its dose are critical in WM performance and for the tuning of prefrontal activity during WM. During the executive (updating) process of WM, dopamine is released from the striatal area; this activity is believed to be critical for WM (Bäckman et al. 2011). The two of main dopaminergic systems in the brain, the nigrostriatal system (in which neurons project to the striatum from the substantia nigra) and the mesocortical system [in which neurons project to the prefrontal cortex and anterior cingulate cortex (ACC) from the ventral tegmental area] (Carlson 2001) are thought to be important for WM (Landau et al. 2009). In the prefrontal cortex, in particular, the dopaminergic function of the dorsolateral prefrontal cortex (DLPFC) is critical for WM (Abi-Dargham et al. 2002).

Previous studies have also shown the effects of WM training (WMT) on psychological measures and neural systems (Klingberg 2010; Takeuchi et al. 2010a). WMT can improve WM performance as well as inhibition/attention measures (Melby-Lervåg and Hulme 2012). In addition, with regard to neural systems, changes in brain activity and gray matter structures in parts of the dopaminergic systems (DLPFC; striatum) following WMT have been demonstrated (Takeuchi et al. 2010a, 2013a). Furthermore, changes in dopamine D1 receptor density in DLPFC, posterior parietal cortex, and other prefrontal cortical areas have been demonstrated after WMT (Klingberg 2010). WMT that involves updating has been shown to reduce transient changes in the binding potentials of D2 receptors in the striatum compared with a control task, which was interpreted as an increase in striatal dopamine release during updating (Bäckman et al. 2011).

Mean diffusivity (MD), measured by diffusion tensor imaging (DTI), is the rate of diffusivity and a direction-independent measure of the average diffusivity reflecting water motility in a voxel. Recently, MD in the dopaminergic system’s areas (MDDS) has been shown to be sensitive to the pathology of dopaminergic systems (Parkinson disease’s) that cannot be detected by other magnetic resonance imaging (MRI) measures such as brain structure volume, a measure sensitive to iron deposition, or fractional anisotropy measures of DTI (Péran et al. 2010). MDDS has been shown to be more accurate in dissociating the pathology of dopaminergic systems than PET measures of dopamine receptor binding (Seppi et al. 2004). It can also detect the neural plasticity caused by medication with dopamine agonists to manage this pathology (Razek et al. 2011). MDDS was also robustly associated with the motivational state and novelty seeking (Takeuchi and Kawashima 2013) which are strongly associated with the function of the dopaminergic system (Bódi et al. 2009; Kaasinen et al. 2004; Kaplan and Oudeyer 2007; Schinka et al. 2002; Suhara et al. 2001; Tomer and Aharon-Peretz 2004). Generally, MD measures in the brain have been considered to reflect the microstructural integrity of brain tissue (Takeuchi et al. 2011a). In addition, MD is considered to detect tissue changes caused by neural plasticity such as astrocyte swelling, synaptic changes, dendritic spine changes, and angiogenesis; therefore, MD can detect short-term as well as long-term neural plasticity (Johansen-Berg et al. 2012; Sagi et al. 2012).

Thus, MD is a sensitive and unique neuroimaging tool that can detect neural plasticity, especially in the areas of dopaminergic system. However, the effects of WMT on MD are not yet known. In the present study, we investigated the effects of WMT on MD.

Young adult subjects were divided into two groups: one group underwent 4-week WMT and the other group did not. Before and after the intervention or no-intervention periods, the subjects underwent scanning sessions including DWI, based on which MD was calculated. Using preprocessing and analysis, we removed the effects of cerebral spinal fluid (CSF) from MD and subsequently focused on MD in the subcortical areas where CSF, which substantially impacts MD, can be clearly separated from neural tissue. Considering the plasticity of the cognitive functions caused by WMT (Takeuchi et al. 2010a) and the critical roles played by WM in higher-order cognitive functions (Baddeley 2003), it is important to elucidate the extent of the plasticity of MD, which can provide unique information, caused by WMT. We hypothesized that WMT alters MD of the areas of the mesocortical system that play key roles in WM and of the areas of the nigrostriatal system, given their critical roles in WM (as described above), as well as the unique sensitivity of MD in the areas of the dopaminergic system. As the unique plasticity of MD and the complex mechanisms of plasticity brought by WMT (McNab et al. 2009; Takeuchi et al. 2011b) remain mostly unknown, WMT may alter MD in either direction; therefore, we did not propose a definitive hypothesis regarding this matter.

## Materials and methods

### Subjects

In this present study, we analyzed data from 51 healthy, right-handed university students who completed the experimental intervention as planned and participated in our previous study, analyzing resting-state fMRI and cerebral blood flow data (Takeuchi et al. 2013a). Sixty-one subjects participated in the study as the WMT and control groups (Takeuchi et al. 2013a); however, one subject in the control group could not participate in the planned post-MRI experiment (Takeuchi et al. 2013c). Among the 60 subjects analyzed in the previous study, we removed data of nine subjects in the present study because their DWI data were not obtained in the same sequence as other subjects. They had normal vision and no history of neurological or psychiatric illness, which was assessed using a routine questionnaire. Handedness was evaluated using the Edinburgh handedness inventory (Oldfield 1971). Subjects were assigned to one of the groups in a random manner. For more details, see our previous study (Takeuchi et al. 2013a). The WM-training group consisted of 34 participants (21 men and 13 women) and the mean age of the subjects of the no-intervention group was 21.0 years [standard deviation (SD) 1.6]. The no-intervention group consisted of 17 participants (13 men and 4 women) and the mean age of the subjects of the no-intervention group was 21.2 years [standard deviation (SD), 2.4]. The WM-training and control groups did not differ significantly (*P* > 0.1, two-tailed *t* tests) in age, sex, or scores on the general intelligence (Raven’s advanced progressive matrix test: Raven 1998) and WM measures (visuospatial WM and digit span tasks) (Takeuchi et al. 2013a). In accordance with the Declaration of Helsinki (World Medical Association 1991), written informed consent was obtained from each subject. This study was approved by the Ethics Committee of Tohoku University.

### Procedure

As described in our previous study (Takeuchi et al. 2013a), the WMT program consisted of computerized, in-house developed Borland C++ programs comprising four computerized tasks. Subjects undertook approximately 4 weeks (27 days) of training (each day, 20–60 min in most cases). However, the total training time depended on the level and time between trials. The subjects used the program provided to them on their personal computers. They were recommended to perform the WMT tasks every day; two training sessions for a week were conducted in the laboratory. More details were described in our previous study (Takeuchi et al. 2013a). We used a control group of no intervention and subjects in the no-intervention group just participated in pre and post experiments based on (1) the recommendation of the previous review (Takeuchi et al. 2010a), (2) the lack of effects in the active control intervention in any measure in our previous study (Takeuchi et al. 2011b), in which sample characteristics and experimental settings are similar, and (3) overwhelming number of studies that failed to show the effects of active control groups (Brown et al. 2009; Clark et al. 1997; Mahncke et al. 2006) as opposed to widely held beliefs. Further, sometimes, it seems the active control groups become inappropriate as control groups due to the specific effects of active control training on measures where they are not supposed to have specific effects (Takeuchi et al. 2010a). The use of a passive control group instead of active control group or study designs without control groups (which is followed by the within-training group analysis with training-related variables) are gold standard procedures for imaging studies of cognitive training (Takeuchi et al. 2010a). In particular, it should be noted that the previous studies have never shown that the well-controlled active control interventions affect brain structural properties in the experimental settings used in the present study (Sagi et al. 2012; Takeuchi et al. 2011b). To have both the active control group and no-intervention group is ideal (Takeuchi et al. 2010a), but usually suboptimal from the statistical perspective (Takeuchi et al. 2010a).

### Training tasks

As described in our previous study (Takeuchi et al. 2013a), four WMT tasks were presented during each training session. In all training tasks, difficulties (number of items to be remembered) were modulated based on subjects’ performance. Four WMT tasks are as follows: (1) a visuospatial WM task, (2) an auditory backward operation span task, (3) a dual WM task, and (4) a dual N-back task.

(1) In the visuospatial WM task, circles are presented one at a time (1/s rate) in an interface where ten squares are irregularly distributed (circles are presented in one of these squares). After stimuli presentation, the subjects indicate the location and order of the presented stimuli by clicking on a computer screen with a mouse. (2) In an auditory backward operation span task, pairs of single digits (0–9) are verbally presented at a rate of 1 pair/3 s. Within this 3 s period, one digit is presented per second, but in the final second, no stimuli are presented. Therefore, four pairs of digits would be presented as follows: 1, 3, no stimulus; 4, 9, no stimulus; 3, 7, no stimulus; and 2, 5, no stimulus. The subjects had to remember the value, which was the sum of the presented pairs of digits and the order in which they were presented (in the above example, they would need to remember 4, 3, 0, 7). After the presentation, the subjects had to repeat the sequence by pressing numbered buttons on the screen in the reverse order (7, 0, 3, 4 in the above example). (3) In the dual WM task, which is similar to the task implemented in the previous study (Dash et al. 2010), the subjects had to concurrently perform a visuospatial WM task and an auditory digit span task. In that study, circles were presented one at a time. with a 1/3 s rate in the same interface used for task (1) After stimuli presentation, the subjects indicate the location and order of the presented stimuli by clicking on a computer screen with a mouse. One digit (0–9) was verbally and simultaneously presented with the circle presentation. After stimuli presentation, the subjects indicated the digits and the order of the stimuli by pressing numbered buttons on the screen in the order they were presented. The subjects could perform either task first. (4) In a dual WM task, which is similar to the task implemented in a previous study of WMT (Jaeggi et al. 2008), squares at eight different locations were sequentially presented on a computer screen at a 3 s rate (stimulus length, 500 ms; inter-stimulus interval, 2,500 ms). Simultaneously with the presentation of the squares, one of eight consonants is sequentially presented using headphones. A response was required whenever one of the presented stimuli matched the stimuli previously presented at (*n*) positions in the sequence. Additional details regarding the practical aspects of the task procedures and training are described in our previous study (Takeuchi et al. 2013a). The amount of the training time for each task is not necessarily exactly equal among four tasks. The task order was always fixed in this order [(1)–(4)]. The same task was not used for the assessment prior to the intervention period.

### Image acquisition and analysis

MRI data acquisition was conducted using a 3T Philips Achieva scanner. Diffusion-weighted data were acquired using a spin-echo EPI sequence (TR = 10,293 ms, TE = 55 ms, FOV = 22.4 cm, 2 × 2 × 2 mm^3^ voxels, 60 slices, SENSE reduction factor = 2, number of acquisitions = 1). The diffusion weighting was isotropically distributed along 32 directions (*b* value = 1,000 s/mm^*2*^). Additionally, three images with no diffusion weighting (*b* value = 0 s/mm^2^) (*b* = 0 images), were acquired using a spin-echo EPI sequence (TR = 10293 ms, TE = 55 ms, FOV = 22.4 cm, 2 × 2 × 2 mm^3^ voxels, 60 slices). From the collected images, MD maps were calculated (Takeuchi et al. 2011a).

Subjects completed the fMRI paradigms of the N-back WM task (2-back WM condition and 0-back control condition) before and after training. Details relating to these N-back fMRI paradigms can be found in our previous report (Takeuchi et al. 2011e). We used the pre-training fMRI data of subjects in the WMT and control groups to construct the regions of interest (ROI) for the analyses of diffusion data.

### Preprocessing and individual-level statistical analysis of imaging data

Preprocessing of imaging data were performed using SPM8 implemented in matlab. Pre- and post-training MD maps were independently normalized. We avoided co-registration and co-normalization procedures (which use the same normalization parameters for both the pre- and post-images) of pre- and post-images, including registration of mean images to pre- and post-images, because of concerns of possible bias or problems occurring when structural properties were substantially different between pre- and post-images (Thomas and Baker 2012).

Pre- and post-scan MD images were segmented and normalized using a previously described diffeomorphic anatomical registration through exponentiated lie algebra (DARTEL)-based method (Takeuchi et al. 2013b), which provided images of 1.5 × 1.5 × 1.5 mm^3^ voxels. Then, from the pre- and post-intervention normalized images of normalized (1) MD map, (2) regional gray matter density (rGMD) map, and (3) regional cerebral spinal fluid map density (rCSF) map, areas that are not strongly likely to be gray matter nor white matter in our custom template (defined by “gray matter tissue probability + white matter tissue probability <0.99”) were removed [to exclude the strong effects of cerebral spinal fluid (CSF) on MD]. Subsequently, these images were smoothed by convolving them with an isotropic Gaussian kernel of 10 mm full width at half maximum. These images were forwarded to the group analysis described below.

BOLD image data of N-back fMRI tasks were co-registered to diffusion data, as described previously (Takeuchi et al. 2011c), and then normalized using the same normalization parameter as that used for diffusion data.

### Statistical group-level analysis of imaging data

In the group-level imaging analysis, we tested for group-wise differences in MD after WMT and subsequently performed voxel-wise one-way ANCOVAs. The dependent variables were the MD values for the post-scan at each voxel, whereas the independent variables were the MD values for the pre-scan at each voxel and the rGMD and rCSFD values for pre- and post- scans at each voxel. These variables were selected to exclude the effects and the extent of these tissues on MD; however, we did not include white matter density as covariates because tissues were gray matter, white matter, or CSF in the areas relevant to the analysis.

To evaluate the impact of removing the effects of rGMD and rCSFD through ANCOVA, we also ran the analysis without including pre and post rGMD and rCSFD maps as covariates.

We used ANCOVA instead of repeated measures ANOVA to control for the effects of pre-training values. Statistical experts strongly recommend the use of ANCOVA instead of repeated measures ANOVA in this type of study design (Dimitrov et al. 2003). In the case of randomized designs, the purpose of ANCOVA is to reduce error variance, whereas in the case of non-randomized designs (or of analyses involving substantial pre-existing group differences), ANCOVA is used to adjust the post-test means for pre-test differences among groups (Dimitrov et al. 2003). One might recommend that the differences in values between pre- and post-training measures be used instead of the post-training values. However, in fact, when the pre-training values are included as covariates, the two analyses return the same statistical value.

This analysis was performed using biological parametrical mapping (Casanova et al. 2007) implemented in SPM5 and those representing pre- and post-intervention MD, rGMD, and rCSFD values. Note, since the control group did not engage in training and there are no variables of amount of training in the control group, we cannot take the effect of amount of training into account in the comparisons of WMT versus control group.

Regions with significance were inferred using cluster-level statistics (Friston et al. 1996). Only clusters with *P* < 0.05, after correction for multiple comparisons at cluster size with a voxel-level cluster-determining threshold of *P* < 0.005 uncorrected, were considered statistically significant in this analysis. This voxel-level cluster-determining threshold has been used in the previous studies (Takeuchi et al. 2010b, 2011e, 2013a), and the validation study (Hayasaka and Nichols 2003) showed this threshold does not cause anti-conservativeness and if anything it seems, it leads to slight conservative results compared with more stringent voxel-level cluster-determining thresholds. Further, ROI analyses have been performed within the areas of the mesocortical system that play a key role in WM, namely DLPFC and the ventral tegmental area, and those of the nigrostriatal system, namely the striatum and substantia nigra, as described in the “Introduction” (Carlson 2001). We constructed a mask image of these areas, and the statistical significance level was set at *P* < 0.05 within these areas, with small volume correction for multiple comparisons (false discovery rate). The mask image for ROIs was constructed by adding mask images for the areas of the bilateral DLPFC around Brodmann area 46, which is central to WM (Osaka and Nishizaki 2000), ACC, bilateral striatum, and midbrain areas, including the ventral tegmental areas and substantia nigra. To construct these images, we used the data of fMRI activation of the 2-back–0-back condition (WM-specific brain activity) before training in the present experiment. Most of the details of the individual analytical methods used here can be found in the previous study (Takeuchi et al. 2011c); however, here, six parameters obtained by rigid body correction of head motion were additionally regressed out by entering these variances into the regressor of the first level analysis, followed by smoothing performed with 10 mm FWHM (same value as the values of the MD data). Subsequently, a whole-brain one-sample *t* test of the 2-back–0-back contrast was performed, and the peak voxels for the dorsal part of ACC to the supplemental motor area (*x*, *y*, *z* = 0, 15, 51), bilateral DLPFC closest to Brodmann area 46 (*x*, *y*, *z* = −36, 51, 18; *x*, *y*, *z* = 42, 30, 33), bilateral striatum (*x*, *y*, *z* = −15, 6, 6; *x*, *y*, *z* = 18, 0, 21), and midbrain areas closest to the ventral tegmental area (Tomasi and Volkow 2012) (*x*, *y*, *z* = 3, −15, 12) were extracted. Then, the masks of 12 mm radial spheres around these voxels of MNI coordinates were created.

### Investigation of the associations between changes in performance on WMT tasks and changes in MD

Next, we investigated whether there was an association between changes in performance on WMT tasks and changes in MD, in which the effects of WMT were observed through simple regression analyses. For this analysis, as described in our previous study (Takeuchi et al. 2013a) performance on WMT tasks for each participant in the first and last three sessions was calculated as follows: (highest level of the visuospatial WM task achieved in the first or last three completed sessions) + (highest level of the auditory backward operation span task achieved in the first or last three completed sessions) + 2 × (highest level of the dual WM task achieved in the first or last three completed sessions) + 2 × (highest level of the dual N-back task achieved in the first or last three completed sessions). Performance on the dual WM task and dual N-back task were multiplied by two because when the level of the dual N-back task was increased by one, the number of stimuli to be remembered was increased by two. An increase in performance on WMT tasks for each participant from the first three sessions to the last three sessions was regarded as an improvement in performance on WMT tasks. The rationale for this calculation using the first/last three sessions (see also the “Training data”) was to obtain stable results since subjects have to perform four tasks in one session and there simply is not sufficient time to ensure stable performance in one session. We did not perform correlation analysis using the amount of WMT as a covariate because in our study, the amount of WMT varied little among subjects (see “Results” for details). Subsequently, we extracted the mean value of the pre- to post-training changes in MD, in each of the significant clusters identified above. Simple regression analyses were then performed to determine the association between the improvement in performance on WMT tasks and the neural changes in each cluster calculated as described above. Here, we employed a one-tailed analysis to investigate the association between an increase in performance on WMT tasks and the mean increase in MD in each cluster because that was our sole hypothesis and interest.

## Results

### Training data

As described in our previous study to investigate the effect of WMT on resting-state functional connectivity (FC), regional cerebral blood flow at rest (resting-rCBF), and regional gray matter volume using data from the same subjects (Takeuchi et al. 2013a), 34 subjects in the WMT group whose data were analyzed in this study completed 25.94 sessions [standard deviation (SD) 2.28] on average and at least 17 sessions during the 27 day intervention period. This indicates that the amount of training in the WMT group is well controlled. Performance of all four trained WM tasks performed during the last three training sessions was significantly improved compared with performance during the first three training sessions (for performance data and statistical values, see Table 1). Details of the training data and training-related changes in performance scores on cognitive tests (such as WM tasks) have been described previously (Takeuchi et al. 2013a).

**Table 1:** The average of all subjects’ highest performances [the N (number of items to be remembered) in the WM tasks in which subjects answered accurately] on WMT tasks among the first and last three training sessions

	First three sessions (items)	Last three sessions (items)
Visuospatial WM task	8.82 ± 0.81	11.48 ± 1.92
Auditory backward operation span task	9 ± 1.44	15.18 ± 3.80
Dual WM task	7.58 ± 0.83	10.03 ± 1.38
Dual N-back task	2.82 ± 0.73	5 ± 1.20

Data obtained from one subject whose final performance data were missing were removed from the calculation of the average in this task

### The effect of WMT on MD

After correcting the effects of pre-training MD and pre- and post-training rGMD and rCSFD, the whole-brain analysis revealed that the WMT group showed significantly greater post-training MD in the anatomical cluster that spread from the right caudate to the right lentiform nucleus (*x*, *y*, *z* = 24, 6, 18; *t* = 4.72; *P* < 0.001; corrected for multiple comparisons at the cluster level with a cluster-determining threshold of *P* < 0.005, uncorrected).

**Fig. 1:**
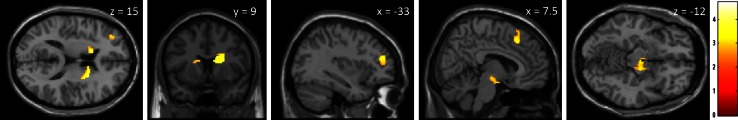
Increase in mean diffusivity in the working memory training (WMT) group compared with the control group. Results are shown with a threshold of *P* < 0.05 and corrected for multiple comparisons at FDR within the regions of interest. Findings were overlaid on a “single-subject T1” SPM image. *Bar* represents the *t* score. The voxel-by-voxel analysis of covariance showed compared with the control intervention (no-intervention), WMT resulted in a significant increase in MD in nigrostriatal and mesocortical areas

Among areas with a strong a priori hypothesis, namely areas of the dopaminergic system, small volume correction was employed and significant effects of WMT were observed in the bilateral caudate, right putamen, left DLPFC, right ACC, right substantia nigra, and ventral tegmental area (Fig. 1). The results of the statistical tests for these comparisons are presented in Table 2.

**Table 2:** Brain regions with significant working memory training (WMT)-related increases in mean diffusivity (MD), as assessed by region of interest (ROI) analysis

Area		*x*	*y*	*z*	*t* score	Corrected *P* value (SVC, FDR)	Cluster size (mm^3^)	Correlation with the improvement in WMT (*P*, *t* , *r* values)*
Caudate/putamen	R	24	6	18	4.72	0.018	2,042	(0.596, −0.244, −0.044)
DLPFC	L	−37.5	43.5	21	3.82	0.022	790	(0.011, 2.398, 0.396)
Caudate	L	−12	13.5	15	3.79	0.022	314	(0.404, 0.246, 0.044)
Substantia nigra (R)/ventral tegmental area		13.5	−10.5	−16.5	3.56	0.024	1,461	(0.807, −0.879, −0.156)
ACC	R	7.5	22.5	48	3.51	0.024	732	(0.009, 2.486, 0.408)
Ventral tegmental area		−1.5	−9	−10.5	3.01	0.031	105	(0.908, −1.359, −0.237)

Statistical values of simple regression analyses that investigated the association between mean change in MD in each cluster and change in performance on WMT tasks

### The effect of WMT on MD without taking into account the effects of rGMD and rCSFD

To evaluate the impact of removing the effects of rGMD and rCSFD through ANCOVAs, we also ran the analysis without including pre and post rGMD and rCSFD maps as covariates.

The whole-brain analysis revealed that the WMT group had a significantly larger MD in the anatomical cluster, which is quite similar to the abovementioned significant cluster of WMT-related increase in MD in the anatomical cluster that spread from the right caudate to the right lentiform nucleus.

SVC was applied among the areas with a strong a priori hypothesis, namely the nigrostriatal area (caudate and putamen) and mesocortical areas (ACC and bilateral DLPFC, which play important roles in working memory, and the ventral tegmental area). This analysis identified regions of significance in the abovementioned ANCOVA using SVC, which took into account the effects of rGMD and rCSFD, yielded significant results in the same contrast in this analysis, with the exception of the area of the left DLPFC (for which we obtained a *P* = 0.08, corrected for FDR). Moreover, in this analysis, the right DLPFC showed significant effects on WMT-related increase in MD (*P* = 0.046, corrected for FDR, *x*, *y*, *z* = 45, 28.5, 30).

These results suggest that the removal of the effects of rGMD and rCSFD did not alter the results substantially [note that in preprocessing, we tried to remove the effects of rCSFD (which can affect MD substantially) as much as possible]; thus, this did not alter our conclusion. However, the statistical value of the left DLPFC was substantially weakened in this analysis and became just a trend. It is not clear whether there is an implication in this subtle change. Nevertheless, in the areas located close to the cortical surface, it may be more difficult to remove the effects of rCSFD completely. Moreover, the possible relative decrease in remaining rCSFD caused by the increase in regional gray matter induced by WMT (Takeuchi et al. 2013a) should lead to a decrease in MD (because CSF has a higher MD); thus, without regressing out those effects, the effect of WMT on an increase in MD may tend to weaken. These mechanisms might explain somewhat the results obtained for the left DLFPC.

### Associations between neural changes and WMT performance changes

Simple regression analyses that tested correlations between improvements in performance on WMT tasks and the amount of MD changes in the significant clusters identified in the abovementioned ANCOVAs were also performed. The results of this analysis showed a significant positive correlation between improvements in performance on WMT tasks and the mean increase in MD in the right ACC and left DLPFC (Table 2).

There were no significant results when the amount of training sessions was used instead of improvements in performance on WMT tasks. The mean increase in MD in the right ACC showed a tendency for a positive correlation with the number of training sessions (*P* = 0.089).

This may be due to a lack of effective variance in the number of training sessions among the WMT groups (see “Training data”) since the amount of training was controlled and we did not include this analysis as a primary method to reveal the effects of WMT.

## Discussion

The present study revealed the effect of WMT on MD in healthy young adults. WMT increased MD in the left DLPFC, right ACC, bilateral caudate, right putamen, right substantia nigra, and ventral tegmental area. The increase in performance on WMT tasks was positively correlated with an increase in MD in the left DLPFC and right ACC. As discussed below, an increase in MD may reflect the number of physiological changes. Thus, the present findings suggest that the WMT-related neural plasticity observed in areas of the mesocortical system that play key roles in WM and in areas of the nigrostriatal system, as well as changes in the left DLPFC and the right ACC, underlie the performance on WMT tasks. These results cannot be explained by the effects of pre-existing group differences in MD, either pre-existing or post-training differences in the distributions of the tissue themselves because we corrected for these effects using ANCOVA.

A prevalent idea suggests that an increase in MDDS after intervention reflects the loss of certain tissue components in normal samples, for the following reasons. MD is often believed to be a measure of overall water content (Moseley et al. 2002). Possible obstacles, such as the presence of fewer or smaller cellular structures (e.g., capillaries, synapses, and macromolecular proteins), may prevent free diffusion of water molecules and may also be expected to cause the value of MD to increase (Ni et al. 2010). Thus, MD can be thought of as a measure of the microstructural integrity (how effective components exist in the tissue) of the brain regardless of the system being investigated, and a wide range of clinical studies of neuronal degeneration have confirmed this (Andreone et al. 2007; Kantarci et al. 2001; Nusbaum et al. 2001). Among normal samples, cognitive learning has been shown to affect MD values in the relevant tissues only after 2 h of training, and together with the results of animal experiments, increases in the number of synaptic vesicles and astrocyte swelling due to increased activity has been suggested to underlie these MD changes (Johansen-Berg et al. 2012; Sagi et al. 2012). Over longer time spans, such as those used in the present study, changes in other physiological mechanisms, such as dendritic sprouting and angiogenesis, may affect MD (Johansen-Berg et al. 2012). Therefore, although there is a wide range of possible mechanisms, these generally considered mechanisms, whether functional (such as activity) or structural (such as synaptic changes), suggest that an increase in MD values in the normal sample represents a certain loss of the components of the tissue system. This interpretation of cognitive intervention-related changes in MD has been proposed in recent studies (Abe et al. 2014; Johansen-Berg et al. 2012; Sagi et al. 2012). However, our previous study, which was performed using a sample that overlapped with the present one, reported WMT-related regional gray matter volume increases in areas that included the left DLFPC, ACC, and left caudate (Takeuchi et al. 2013a). Moreover, animal experiments showed that an rGMV increase followed by a cognitive intervention reflects neuronal remodeling, including an increase in synapses (Lerch et al. 2011). These previous findings indicate that the present WMT-related increase in MD may not be mediated by a synaptic decrease, among the possible causes that were suggested above.

However, other factors may explain the changes in MD that occur after WMT. One is the increase in the resting-state cerebral flow in the areas reported here. It is known that MD will also increase based on the increase in cerebral blood flow (Jin and Kim 2008; Song et al. 2002). Thus, training-induced plasticity might lead to an increase in the resting-state cerebral blood flow in the areas reported here, which in turn might increase MD. This idea is congruent with the present findings, which showed that the increase in MD was associated with improvement of the performance on WMT tasks in the left DLPFC and ACC, thus suggesting that the increase in MD caused by WMT was somehow an adaptive change. However, this interpretation is inconsistent with our previous findings, which were obtained using overlapping samples and showed an increase in the resting-state cerebral blood flow only in the right DLPFC (Takeuchi et al. 2013a). In contrast, this area failed to exhibit a significant change in MD in this study. Thus, this interpretation is not definitive, although the lack of WMT-related significant resting-state cerebral blood flow in ACC or left DLPFC or subcortical areas may be explained by the relatively low spatial resolution and fragility toward motion (Takeuchi et al. 2011d). Finally, although this is a highly speculative idea, MDDS can also be related to the functioning of areas of the dopaminergic system as a result of strong associations between metals and a number of dopaminergic functions (McCann and Ames 2007). The MD values in gray matter are known to be lowered by metals including iron and copper with paramagnetic properties because of the effects of paramagnetic properties on MRI (Sener 2002). Furthermore, some metals, such as iron and copper, have long been suggested to play essential roles in the dopaminergic system, and, these metals are required for dopamine production (McCann and Ames 2007). On the other hand, previously, iron has been shown to accumulate in dopamine neurovesicles (Ortega et al. 2007). In addition, the inhibition of dopamine synthesis results in decreased vesicular storage of iron (Ortega et al. 2007). Thus, the lowering in the level of dopamine induced by WMT in tissues may have increased MD signal intensities in the areas reported here. However, this idea is consistent with the previous findings of the specific sensitivity of MD toward pathology of the dopamine system and its plasticity, as well as the psychological state and trait associated with the function of dopamine, as described in the “Introduction”. Nevertheless, this idea is not supported by the experimental findings, unlike other possibilities.

Thus, as discussed above, an increase in MD may reflect several physiological processes, and we cannot conclude definitely on which processes contributed to the increase in MD observed after WMT. This difficulty in determining the physiological changes that underlie the signal change observed in the images is similar to the case of regional gray matter volume images (Takeuchi et al. 2011b, 2014). Nonetheless, as discussed above, the present result of WMT-related increase in MD is not congruent with our previously reported WMT-related regional gray matter volume increase in a similar area, if we assume the same physiological mechanisms (such as synaptic changes) underlie both changes. Furthermore, recent previous studies reported the sensitivity of MD to detect the neural plasticity that occurs soon after the intervention (Abe et al. 2014; Johansen-Berg et al. 2012). Thus, the results of the present study, together with those of previous studies, suggest the applicability of the unique properties of MD to investigate and identify regional neural plasticity.

In the present study, many areas of the gray matter showed changes in MD. These changes observed in gray matter are highly consistent with the results of previous studies that investigated the effects of intervention on MD (Abe et al. 2014; Razek et al. 2011; Sagi et al. 2012). Furthermore, two of these studies reported an increase in MD in the gray matter (Abe et al. 2014; Razek et al. 2011). As described above, changes in synapse or synaptic vesicles, astrocytes, dendritic sprouting, and angiogenesis have been demonstrated or considered as the causes of these changes in MD. These changes occur mainly in the gray matter. Thus, the fact that the present findings were observed in many gray matter areas is consistent with those micro-level findings or suggestions.

The areas of significant MD change overlapped two of the main dopaminergic systems in the brain; the nigrostriatal system, and the mesocortical system (Carlson 2001). Neurons in the nigrostriatal system project to the striatum from the substantia nigra (Carlson 2001) and this system plays a key role in motor control (Carlson 2001). Involvement of this system has been implicated with other higher-order cognitive functions including filtering information flow to the frontal lobe, updating, or motivation (Dahlin et al. 2008; McNab and Klingberg 2007; Takeuchi et al. 2012). Finally, neurons in the mesocortical system projects to the prefrontal cortex and ACC from the tegmental ventral area, and are involved in problem solving, working memory, and other higher-order cognitive functions (Carlson 2001; Schweimer and Hauber 2006). The neural changes observed in areas that are crucial to WM may contribute to the increase in cognitive functions induced by WMT.

## References

[CR1] Aalto S, Bruck A, Laine M, Nagren K, Rinne JO (2005). Frontal and temporal dopamine release during working memory and attention tasks in healthy humans: a positron emission tomography study using the high-affinity dopamine D2 receptor ligand [11C] FLB 457. J Neurosci.

[CR2] Abe M, Fukuyama H, Mima T (2014). Water diffusion reveals networks that modulate multiregional morphological plasticity after repetitive brain stimulation. Proc Natl Acad Sci.

[CR3] Abi-Dargham A, Mawlawi O, Lombardo I, Gil R, Martinez D, Huang Y, Hwang D-R, Keilp J, Kochan L, Van Heertum R (2002). Prefrontal dopamine D1 receptors and working memory in schizophrenia. J Neurosci.

[CR4] Andreone N, Tansella M, Cerini R, Versace A, Rambaldelli G, Perlini C, Dusi N, Pelizza L, Balestrieri M, Barbui C (2007). Cortical white-matter microstructure in schizophrenia Diffusion imaging study. British J Psychiatry.

[CR5] Bäckman L, Nyberg L, Soveri A, Johansson J, Andersson M, Dahlin E, Neely AS, Virta J, Laine M, Rinne JO (2011). Effects of working-memory training on striatal dopamine release. Science.

[CR6] Baddeley A (2003). Working memory: looking back and looking forward. Nat Rev Neurosci.

[CR7] Bódi N, Kéri S, Nagy H, Moustafa A, Myers CE, Daw N, Dibó G, Takáts A, Bereczki D, Gluck MA (2009). Reward-learning and the novelty-seeking personality: a between-and within-subjects study of the effects of dopamine agonists on young Parkinson’s patients. Brain.

[CR8] Brown AK, Liu-Ambrose T, Tate R, Lord SR (2009). The effect of group-based exercise on cognitive performance and mood in seniors residing in intermediate care and self-care retirement facilities: a randomised controlled trial. Br J Sports Med.

[CR9] Carlson NR (2001). Physiology of behavior.

[CR10] Casanova R, Srikanth R, Baer A, Laurienti PJ, Burdette JH, Hayasaka S, Flowers L, Wood F, Maldjian JA (2007). Biological parametric mapping: a statistical toolbox for multimodality brain image analysis. Neuroimage.

[CR11] Clark F, Azen S, Zemke R, Jackson J, Carlson M, Mandel D, Hay J, Josephson K, Cherry B, Hessel C (1997). Occupational therapy for independent-living older adults. A randomized controlled trial. J Am Med Assoc.

[CR12] Dahlin E, Neely AS, Larsson A, Backman L, Nyberg L (2008). Transfer of learning after updating training mediated by the striatum. Science.

[CR13] Dash D, McNab F, Klingberg T (2010) Effect of dual working memory training on a reasoning task‐ an fMRI study. In: 16th Annual meeting of the organization for human brain mapping, Barcelona, Spain

[CR14] Dimitrov DM, Rumrill J, Phillip D (2003). Pretest-posttest designs and measurement of change. Work.

[CR15] Friston KJ, Holmes A, Poline JB, Price CJ, Frith CD (1996). Detecting activations in PET and fMRI: levels of inference and power. Neuroimage.

[CR16] Hayasaka S, Nichols TE (2003). Validating cluster size inference: random field and permutation methods. Neuroimage.

[CR17] Jaeggi SM, Buschkuehl M, Jonides J, Perrig WJ (2008). Improving fluid intelligence with training on working memory. Proc Natl Acad Sci.

[CR18] Jin T, Kim S-G (2008). Functional changes of apparent diffusion coefficient during visual stimulation investigated by diffusion-weighted gradient-echo fMRI. Neuroimage.

[CR19] Johansen-Berg H, Baptista CS, Thomas AG (2012). Human structural plasticity at record speed. Neuron.

[CR20] Kaasinen V, Aalto S, Någren K, Rinne JO (2004). Insular dopamine D2 receptors and novelty seeking personality in Parkinson’s disease. Mov Disord.

[CR21] Kantarci K, Jack CR, Xu YC, Campeau NG, O’Brien PC, Smith GE, Ivnik RJ, Boeve BF, Kokmen E, Tangalos EG (2001). Mild cognitive impairment and alzheimer disease: regional diffusivity of water1. Radiology.

[CR22] Kaplan F, Oudeyer P-Y (2007). In search of the neural circuits of intrinsic motivation. Front Neurosci.

[CR23] Klingberg T (2010). Training and plasticity of working memory. Trends Cogn Sci.

[CR24] Landau SM, Lal R, O’Neil JP, Baker S, Jagust WJ (2009). Striatal dopamine and working memory. Cereb Cortex.

[CR25] Lerch JP, Yiu AP, Martinez-Canabal A, Pekar T, Bohbot VD, Frankland PW, Henkelman RM, Josselyn SA, Sled JG (2011). Maze training in mice induces MRI-detectable brain shape changes specific to the type of learning. Neuroimage.

[CR26] Mahncke HW, Connor BB, Appelman J, Ahsanuddin ON, Hardy JL, Wood RA, Joyce NM, Boniske T, Atkins SM, Merzenich MM (2006). Memory enhancement in healthy older adults using a brain plasticity-based training program: a randomized, controlled study. Proc Natl Acad Sci.

[CR27] McCann JC, Ames BN (2007). An overview of evidence for a causal relation between iron deficiency during development and deficits in cognitive or behavioral function. Am J Clin Nutr.

[CR28] McNab F, Klingberg T (2007). Prefrontal cortex and basal ganglia control access to working memory. Nat Neurosci.

[CR29] McNab F, Varrone A, Farde L, Jucaite A, Bystritsky P, Forssberg H, Klingberg T (2009). Changes in cortical dopamine D1 receptor binding associated with cognitive training. Science.

[CR30] Melby-Lervåg M, Hulme C (2012). Is working memory training effective? A meta-analytic review. Dev Psychol.

[CR31] Moseley M, Bammer R, Illes J (2002). Diffusion-tensor imaging of cognitive performance. Brain Cogn.

[CR32] Ni J, Chen S, Liu J, Huang G, Shen T, Chen X (2010). Regional diffusion changes of cerebral grey matter during normal aging–A fluid-inversion prepared diffusion imaging study. Eur J Radiol.

[CR33] Nusbaum AO, Tang CY, Buchsbaum MS, Wei TC, Atlas SW (2001). Regional and global changes in cerebral diffusion with normal aging. Am J Neuroradiol.

[CR34] Oldfield RC (1971). The assessment and analysis of handedness: the Edinburgh inventory. Neuropsychologia.

[CR35] Ortega R, Cloetens P, Devès G, Carmona A, Bohic S (2007). Iron storage within dopamine neurovesicles revealed by chemical nano-imaging. PLoS One.

[CR36] Osaka M, Nishizaki Y (2000). Brain and working memory (in Japanese).

[CR37] Péran P, Cherubini A, Assogna F, Piras F, Quattrocchi C, Peppe A, Celsis P, Rascol O, Démonet J-F, Stefani A (2010). Magnetic resonance imaging markers of Parkinson’s disease nigrostriatal signature. Brain.

[CR38] Raven J (1998). Manual for Raven’s progressive matrices and vocabulary scales.

[CR39] Razek AA, Elmongy A, Hazem M, Zakareyia S, Gabr W (2011). Idiopathic Parkinson disease effect of levodopa on apparent diffusion coefficient value of the brain. Acad Radiol.

[CR40] Sagi Y, Tavor I, Hofstetter S, Tzur-Moryosef S, Blumenfeld-Katzir T, Assaf Y (2012). Learning in the fast lane: new insights into neuroplasticity. Neuron.

[CR41] Schinka J, Letsch E, Crawford F (2002). DRD4 and novelty seeking: results of meta-analyses. Am J Med Genet.

[CR42] Schweimer J, Hauber W (2006). Dopamine D1 receptors in the anterior cingulate cortex regulate effort-based decision making. Learn Mem.

[CR43] Sener R (2002). Echo-planar and gradient-echo diffusion MRI of normal brain iron in the globus pallidus. Clin Imaging.

[CR44] Seppi K, Schocke MF, Donnemiller E, Esterhammer R, Kremser C, Scherfler C, Diem A, Jaschke W, Wenning GK, Poewe W (2004). Comparison of diffusion-weighted imaging and [123I] IBZM-SPECT for the differentiation of patients with the Parkinson variant of multiple system atrophy from those with Parkinson’s disease. Mov Disord.

[CR45] Song AW, Woldorff MG, Gangstead S, Mangun GR, McCarthy G (2002). Enhanced spatial localization of neuronal activation using simultaneous apparent-diffusion-coefficient and blood-oxygenation functional magnetic resonance imaging. Neuroimage.

[CR46] Suhara T, Yasuno F, Sudo Y, Yamamoto M, Inoue M, Okubo Y, Suzuki K (2001). Dopamine D2 receptors in the insular cortex and the personality trait of novelty seeking. Neuroimage.

[CR47] Takeuchi H, Kawashima R (2013) Neural bases of individual differences of creativity measured by the divergent thinking test (symposium). In: Association for psychological science 25th annual convention. Washington DC, USA

[CR48] Takeuchi H, Taki Y, Kawashima R (2010). Effects of working memory training on cognitive functions and neural systems. Rev Neurosci.

[CR49] Takeuchi H, Sekiguchi A, Taki Y, Yokoyama S, Yomogida Y, Komuro N, Yamanouchi T, Suzuki S, Kawashima R (2010). Training of working memory impacts structural connectivity. J Neurosci.

[CR50] Takeuchi H, Taki Y, Sassa Y, Hashizume H, Sekiguchi A, Fukushima A, Kawashima R (2011). Verbal working memory performance correlates with regional white matter structures in the fronto-parietal regions. Neuropsychologia.

[CR51] Takeuchi H, Taki Y, Sassa Y, Hashizume H, Sekiguchi A, Fukushima A, Kawashima R (2011). Working memory training using mental calculation impacts regional gray matter of the frontal and parietal regions. PLoS One.

[CR52] Takeuchi H, Taki Y, Hashizume H, Sassa Y, Nagase T, Nouchi R, Kawashima R (2011). Effects of training of processing speed on neural systems. J Neurosci.

[CR53] Takeuchi H, Taki Y, Hashizume H, Sassa Y, Nagase T, Nouchi R, Kawashima R (2011). Cerebral blood flow during rest associates with general intelligence and creativity. PLoS One.

[CR54] Takeuchi H, Taki Y, Hashizume H, Sassa Y, Nagase T, Nouchi R, Kawashima R (2011). Failing to deactivate: the association between brain activity during a working memory task and creativity. Neuroimage.

[CR55] Takeuchi H, Taki Y, Nouchi R, Sekiguchi A, Kotozaki Y, Miyauchi C, Yokoyama R, Iizuka K, Hashizume H, Nakagawa S (2012) Regional gray matter density is associated with achievement motivation: evidence from voxel-based morphometry. Brain Struct Fun Epub ahead of print 10.1007/s00429-012-0485-3 PMC3889816 23212300

[CR56] Takeuchi H, Taki Y, Nouchi R, Hashizume H, Sekiguchi A, Kotozaki Y, Nakagawa S, Miyauchi CM, Sassa Y, Kawashima R (2013). Effects of working memory-training on functional connectivity and cerebral blood flow during rest. Cortex.

[CR57] Takeuchi H, Taki Y, Thyreau B, Sassa Y, Hashizume H, Sekiguchi A, Nagase T, Nouchi R, Fukushima A, Kawashima R (2013b) White matter structures associated with empathizing and systemizing in young adults. Neuroimage Epub ahead of print 10.1016/j.neuroimage.2013.04.004 23578577

[CR58] Takeuchi H, Taki Y, Nouchi R, Hashizume H, Sekiguchi A, Kotozaki Y, Nakagawa S, Miyauchi CM, Sassa Y, Kawashima R (2013c) Effects of Multitasking-Training on Gray Matter Structure and Resting State Neural Mechanisms. Human Brain Mapping Epub ahead of publication 10.1002/hbm.22427 PMC4216411 24343872

[CR59] Takeuchi H, Taki Y, Sassa Y, Hashizume H, Sekiguchi A, Fukushima A, Kawashima R (2014). Regional gray matter volume is associated with empathizing and systemizing in young adults. PLoS One.

[CR60] Thomas C, Baker CI (2012). Teaching an adult brain new tricks: a critical review of evidence for training-dependent structural plasticity in humans. Neuroimage.

[CR61] Tomasi D, Volkow ND (2012) Functional connectivity of substantia nigra and ventral tegmental area: maturation during adolescence and effects of ADHD. Cerebral Cortex First published online 10.1093/cercor/bhs382 PMC3948497 23242198

[CR62] Tomer R, Aharon-Peretz J (2004). Novelty seeking and harm avoidance in Parkinson’s disease: effects of asymmetric dopamine deficiency. J Neurol Neurosurg Psychiatry.

[CR63] Wechsler D (1997). WAIS-III administration and scoring manual.

[CR64] World Medical Association (1991) Declaration of Helsinki. Law Med Health Care 19:264–265 11642954

